# A Focused Comparative Review of Innovative Therapeutics Across Autoimmune and Chronic Inflammatory Diseases

**DOI:** 10.3390/life16050736

**Published:** 2026-04-28

**Authors:** Harisa Hibić Kaknjašević, Emina Dervišević, Almir Fajkić, Azra Hodžić, Alexander Chupin, Emina Karahmet Sher

**Affiliations:** 1 Department of Maxillofacial Surgery, Cantonal Hospital Zenica, 72000 Zenica, Bosnia and Herzegovina; 2 International Society of Engineering Science and Technology, Nottingham NG11 8BQ, UK; 3 Department of Forensic Medicine, Faculty of Medicine, University of Sarajevo, 71000 Sarajevo, Bosnia and Herzegovina; 4 Department of Pathophysiology, Faculty of Medicine, University of Sarajevo, 71000 Sarajevo, Bosnia and Herzegovina; 5 Department of International Economic Relations, Peoples’ Friendship University of Russia (RUDN University), 117198 Moscow, Russia; 6 Leicester School of Pharmacy, Faculty of Health and Life Sciences, De Montfort University, Leicester LE1 9BH, UK

**Keywords:** autoimmune diseases, chronic inflammatory diseases, immune-mediated disorders, biologics, JAK inhibitors, precision medicine, nanotechnology-based drug delivery

## Abstract

Chronic inflammatory diseases and autoimmune diseases are overlapping but distinct immune-mediated disorders that represent a growing worldwide health concern, characterised by persistent inflammation, tissue damage, and progressive organ dysfunction. In the United States alone, more than $180 billion is spent annually on managing these conditions, yet fewer than 10% of patients achieve long-term remission. These figures highlight the limitations of conventional therapies, which often control symptoms rather than adequately modify the underlying disease process. This review provides a focused and comparative overview of emerging therapeutic strategies across representative immune-mediated disorders, with particular emphasis on mesenchymal stem cells, Janus kinase-signal transducer and activator of transcription (JAK-STAT) inhibitors, chimeric antigen receptor T-cell therapies, therapeutic vaccines, microbiome-modulating interventions, and nanotechnology-based drug delivery systems. In parallel, artificial intelligence (AI) is increasingly contributing to biomarker discovery, drug repurposing, and treatment stratification, thereby supporting the development of predictive and personalised medicine. Overall, these advances support a shift toward mechanism-based, multimodal, and more durable treatment strategies, although further clinical validation remains necessary.

## 1. Introduction

During the host defence response, our body uses one of its mechanisms, called inflammation. Prolonged, low-grade defence, which can last for years, is chronic inflammation, in contrast to acute inflammation, which is fast and lasts for several days [1]. Chronic inflammation results in chronic inflammatory diseases (CIDs), while autoimmune diseases (AIDs) represent a distinct but overlapping group of immune-mediated disorders in which, following immune system dysfunction, the body fights its own tissues [2]. Inflammatory diseases are a global and growing health problem and are among the leading causes of morbidity and mortality, where their great heterogeneity and broad definitions place substantial pressure on the healthcare system [3]. The spectrum of inflammatory diseases continues to expand, with genetic predisposition and environmental factors playing key roles in their development [4]. Particular attention has been given to infections [5], sleep disturbances [6], stress [7], air pollution [8], climate change, and, most notably, changes in diet and gut microbiome. Autoimmune diseases primarily impair patients’ health status and quality of life, and raise public and private costs. We must intensify our efforts in research, diagnosis, and prevention of this burden [4].

CIDs are a major global health concern, with substantial differences in prevalence across various regions and populations, as given in Table 1. Psoriasis affects approximately 125 million people worldwide, with prevalence rates ranging from 0.33% to 0.6% across different racial populations [9]. Systemic lupus erythematosus (SLE) impacts around 3.4 million individuals globally, with women constituting approximately 90% of affected cases. In the United States, as many as 3.1 million adults (1.3%) are affected by inflammatory bowel disease (IBD) [10], with a similar prevalence in Europe of 3 million people (0.3%) [11]. The disease is also on the rise in industrialised parts of Asia [12]. These examples illustrate the global burden and heterogeneity of representative CIDs and AIDs discussed in this review.

Autoimmune diseases predominantly affect women, who account for about 80% of diagnosed cases. The female-to-male prevalence ratio is particularly high in SLE and rheumatoid arthritis (RA), due to a combination of genetic, hormonal changes (in puberty, pregnancy, and menopause) and immunological factors. Studies have shown a higher prevalence of SLE in women of reproductive age (20–40 years) and in women of Black and Asian ancestry [22]. A recent cohort study conducted in the UK showed that, between 2000 and 2019, the incidence of autoimmune diseases increased by 4%, with the highest rises observed in coeliac disease, SS, and Graves’ disease. An estimated 10.2% of the population has autoimmune diseases, with a higher prevalence in women (13.1%) compared to men (7.4%) [23]. Chronic inflammatory and autoimmune diseases present significant challenges due to the inadequate therapeutic response in many patients treated with conventional therapies, including immunosuppressants and biologics. Despite advancements in treatment, many patients still do not reach sustained remission, and some eventually become resistant to currently available therapies. To address the unique characteristics of patient subgroups, it is critical to provide individualised treatment. Conventional therapies are largely focused on relieving symptoms rather than adequately modulating the underlying inflammatory process and targeting only a limited number of molecular pathways.

Furthermore, prolonged use of immunosuppressive and biologic medicines is associated with increased risk of infections and malignancies. Emerging strategies, such as nanotechnology-based drug delivery systems, monoclonal antibodies, therapeutic vaccines, and artificial intelligence (AI) in drug discovery, have the potential to improve the current situation. This review aims to contribute to the development of personalised treatment approaches across representative immune-mediated diseases that have the potential to significantly improve outcomes and reshape the future landscape of immune-mediated disease management.

## 2. Mechanisms Underlying Chronic Inflammatory and Autoimmune Conditions

**Figure 1:**
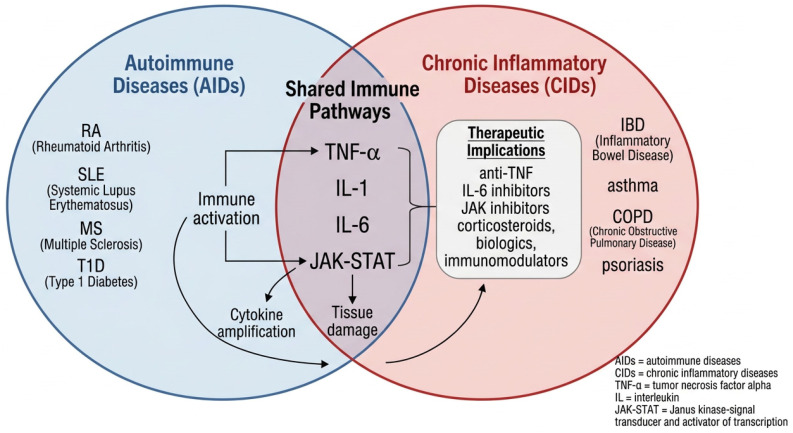
Pathophysiology of Chronic Inflammatory and Autoimmune Diseases [36]. Shared immune pathways are driven by interleukin-mediated signalling, especially IL-1 and IL-6, which amplify inflammatory responses and recruit immune cells. These cytokines sustain chronic tissue inflammation through positive feedback loops and downstream pathways such as JAK-STAT, contributing to both autoimmune and chronic inflammatory disease processes.

Genetics and environmental factors interact in the development of autoimmune diseases, leading to a loss of immunological tolerance and the emergence of autoreactive T and B cells that target their own tissues via cytokines or autoantibodies. This immune imbalance, as a driver of autoimmunity, is shown in Figure 1 [24]. T and B cells, NK cells, dendritic cells, and macrophages produce TNF-α as a mediator of inflammation. It is produced in both membrane-bound and soluble forms—the soluble variant is generated by cleavage by the TNF-α-converting enzyme (TACE) and exerts its effects through TNFR1 and TNFR2 receptors [25]. TNFR1 mediates inflammation, cell death, and immune responses, while TNFR2 regulates immune cell activity. TNFR1 activation triggers three pathways: apoptosis via Caspase 8 and Caspase 3; NF-κB activation for the expression of inflammatory proteins; and MAPK signalling through JNK and AP-1, influencing apoptosis and cell survival [26,27]. Since excessive TNF-α production can lead to various inflammatory diseases, TNF-α blockers have emerged as effective treatments for these conditions. These drugs (etanercept, infliximab, adalimumab, golimumab, and certolizumab) work by preventing the binding of TNF-α to its receptor and promoting apoptosis of cells that produce TNF-α [28].

TNF-α contributes to the pathogenesis of IBD by driving inflammation and promoting the production of IL-1β and IL-6. TNFR1 and TNFR2 play critical roles in regulating disease activity, and TNF-α overproduction impairs mucosal barrier function. IBD, which includes ulcerative colitis (UC) and Crohn’s disease (CD), is presented in this review as a representative chronic inflammatory disease characterised by recurrent inflammation, with a complex aetiology and an imbalance between proinflammatory and anti-inflammatory cytokines [29]. IBD affects approximately 1.5 million people in the United States, 2.2 million in Europe, and hundreds of thousands more globally [30]. The IL-1 family comprises 11 cytokines that mediate systemic and local inflammation and share structural features and receptor-binding mechanisms. It comprises proinflammatory mediators such as IL-1β and IL-18, as well as anti-inflammatory receptor antagonists. IL-1 cytokines act as extracellular soluble factors that bind specific receptors to trigger inflammatory or regulatory responses [31]. IL-1α and IL-1β exert their effects via the IL-1R1/IL-1R3 receptor complex. IL-1β needs activation by caspase-1 to become biologically active, whereas IL-1α can act in its full-length or cleaved form and often serves as an alarmin released during cell damage or death [32].

Multiple mechanisms regulate IL-1 cytokine activity. The receptor antagonist (IL-1Ra) competes with IL-1α and IL-1β for receptor binding without triggering signal activation. Although IL-1R2 binds IL-1β, it lacks a functional domain, thereby preventing signal transduction. Additional reduction in IL-1 activity is achieved through soluble IL-1 receptors that act as decoys. Furthermore, IL-1β, a key proinflammatory cytokine, is produced as an inactive precursor in phagocytes and requires activation within the inflammasome. Once activated, it enhances inflammation by inducing cytokine production, increasing vascular permeability, and recruiting immune cells. IL-1β inhibitors, including monoclonal antibodies and receptor antagonists, help control excessive inflammation in representative immune-mediated diseases [33].

RA is characterised by infiltration of Th1 cells and macrophages, which release TNF-α, a central cytokine in disease progression [34], as shown in Figure 1. Along with IL-1 and IL-6, TNF-α activates synovial fibroblasts, stimulates epidermal hyperplasia, and recruits more inflammatory cells. This cascade leads to the breakdown of collagen and proteoglycans, resulting in cartilage and bone destruction and, ultimately, joint erosion. TNF-α also activates osteoclasts, which contribute to synovial hyperplasia and angiogenesis [35]. The link between IL-1 and RA was first identified in 1977 when Dayer et al. discovered a mononuclear cell factor that stimulated synoviocytes to produce matrix metalloproteases and prostaglandin E2. Subsequent research confirmed IL-1β’s key role in RA pathogenesis. In RA synovium, IL-1β sustains chronic inflammation, primarily produced by monocytes and macrophages, and further amplifies inflammatory responses [36]. In RA synovium, IL-1β sustains chronic inflammation, primarily produced by monocytes and macrophages, and further amplifies inflammatory responses by releasing TNF-α, MCP-1, and IL-8 [31].

The IL-1 family of cytokines also plays a complex role in the pathogenesis of IBD. IL-1β is overexpressed in the inflamed gut of IBD patients, and it is crucial for the differentiation of Th17 cells and the activation of ILC3 cells [37]. The limited efficacy of IL-1β inhibitors in IBD and the absence of intestinal inflammation in autoinflammatory disorders suggest a complex role for IL-1β in gut inflammation. Some IBD patients with CARD8 loss-of-function mutations experience inflammasome activation and IL-1β overproduction, in whom IL-1β inhibitors show strong therapeutic effects. Liu et al. observed increased IL-1β levels in IL-10 knockout mice, even before the development of colitis [38].

Dysregulation of IL-6 is associated with the development of autoimmune diseases such as RA and SLE. TNF-α, IL-1, and coagulation factors stimulate IL-6 production. It affects immune balance by promoting B cell maturation, thereby increasing antibody production, enhancing cytotoxic T cell activity, and raising vascular permeability through complement activation [39,40]. Therapies targeting this cytokine, such as tocilizumab, have shown benefits in controlling cytokine storms and alleviating severe symptoms in selected inflammatory settings and in autoimmune diseases such as SLE and RA. Modulating IL-6 activity may help regulate excessive immune responses and prevent tissue damage in representative immune-mediated diseases [40,41].

## 3. Current Conventional Treatments for Chronic Inflammatory and Autoimmune Diseases

Common medicines used to treat chronic inflammatory and autoimmune diseases are given in Table 2. Some more information on the use and limitations of conventional medicines is provided below.

### 3.1. Corticosteroids

Corticosteroids (e.g., prednisone, prednisolone, methylprednisolone, dexamethasone), as immunomodulatory and anti-inflammatory drugs, bind to their receptors, forming complexes that enter the nucleus and regulate gene expression, thereby increasing or decreasing the levels of inflammatory or anti-inflammatory proteins [47]. As immunomodulators, corticosteroids are commonly used in the treatment of autoimmune diseases, alone or combined with other immunosuppressive drugs [48,49,50]. The therapeutic efficacy and adverse effects of corticosteroids depend on receptor saturation, with higher doses leading to near-complete receptor occupancy and stronger effects [51]. The dose and method of corticosteroid administration depend on the patient’s needs; however, due to potential side effects, their use is carefully assessed [52]. The COBRA study demonstrated that combining glucocorticoids (GC) with methotrexate (MTX) and sulfasalazine (SSZ) reduces radiographic joint damage and disease activity. Although the COBRA strategy showed clinical advantages, the complexity of the treatment regimen and the high initial GC doses are not always applicable in clinical practice. Some studies, such as COBRA-light, used lower initial GC doses and achieved results similar to those obtained with higher initial GC doses [53].

Despite the beneficial clinical effects of corticosteroids, long-term use leads to a range of side effects and serious risks such as cardiovascular disease, toxicity, organ damage and diabetes mellitus [54]. In patients treated with GC for more than six months, osteoporosis may develop in up to 50% of cases [51]. Prolonged use of GC in SLE and RA is linked to dyslipidemia, atherosclerosis, and increased cardiovascular risk, with studies showing higher hypertension rates, especially at doses over 10 mg/day. While low-dose corticosteroids (up to 15 mg/day) are recommended for autoimmune diseases, a recent systematic review shows ongoing uncertainty about high-dose use [54]. A significant knowledge gap remains in understanding the relationship between corticosteroid dosage and therapeutic response. Furthermore, research on the economic burden of corticosteroid use is rare, with only a single study identified in the reviewed literature that specifically addressed this aspect [55].

### 3.2. Nonsteroidal Anti-Inflammatory Drugs

Nonsteroidal anti-inflammatory drugs (NSAIDs) are among the most frequently prescribed medications, mostly for the treatment of pain and inflammation [56]. Although effective in managing chronic inflammation, toxicity to the gastrointestinal and cardiovascular systems, nephrotoxicity, and hepatotoxicity are serious side effects that may occur [57]. The primary mechanism of action of these drugs involves the inhibition of PGHS-1 (COX-1), which is generally constitutive and involved in housekeeping functions, and PGHS-2 (COX-2), which is inducible by inflammatory stimuli and more closely associated with therapeutic anti-inflammatory effects [58]. Due to risks from prolonged use and high doses, the FDA advises using them for a short time and at a low effective dose [59]. To reduce NSAID toxicity while maintaining therapeutic effects, researchers have developed prodrugs that undergo in vivo transformation. Strategies include modifying the carboxyl group to improve selectivity, adding NO- and H_2_S-releasing components for gastroprotection, and developing phospho-NSAIDs for COX-independent action. Although promising in clinical trials, further research is needed on safety and efficacy [60].

### 3.3. Disease-Modifying Antirheumatic Drugs

Therapeutic approaches for autoimmune and chronic inflammatory diseases also include the use of additional immunosuppressive and immunomodulatory medications known as disease-modifying antirheumatic drugs (DMARDs). These drugs are classified into conventional synthetic, biologic, and targeted synthetic types. Conventional synthetic DMARDs—most notably methotrexate, leflunomide, sulfasalazine, and hydroxychloroquine serve as a foundation for treating a wide range of immunological and inflammatory conditions. Their mechanisms of action differ by agent and include inhibition of folate-dependent metabolic pathways, suppression of lymphocyte proliferation, modulation of cytokine production, and interference with antigen presentation and intracellular immune signalling. When these drugs fail to elicit an adequate therapeutic response, treatment is escalated to biologic DMARDs (adalimumab, infliximab, etanercept, etc), which are specifically designed to target key molecules implicated in the pathogenesis of the disease [61,62]. In RA, methotrexate is frequently recommended as the first-line therapy, largely due to its well-established safety profile and affordability, making it especially suitable for long-term use. Sulfasalazine has demonstrated clinical benefits in reducing joint swelling, pain, and morning stiffness in RA patients. Similarly, hydroxychloroquine has proven effective in managing swollen joints and, compared with other conventional DMARDs, offers a favourable safety profile, with a lower risk of serious infections, hepatotoxicity, or renal impairment [13]. DMARDs can be very effective in reducing inflammation, relieving symptoms, and slowing the progression of the disease, especially in diseases such as rheumatoid arthritis. However, the biggest disadvantage of using DMARDs is that the disease is not cured and that treatment is long-term and requires ongoing monitoring [52].

Common side effects of DMARDs include nausea, diarrhoea, stomach pain, skin rash, headache, fatigue, and changes in blood counts and liver enzymes. Another disadvantage of using DMARDs is that, as most are immunosuppressive, there is an increased risk of infections. Biologics and targeted therapies also carry risks of tuberculosis, infusion reactions, and cardiovascular and thrombotic events, depending on the drug used. Another disadvantage of using DMARDs is that not all patients respond equally to the drug, and some patients lose their response over time. This is why the treat-to-target strategy is often employed in the management of patients with rheumatic diseases [52].

## 4. Advances in Targeted Biologic Therapies

### 4.1. TNF-α Inhibitors

TNF-α inhibitors can be used to treat various chronic inflammatory diseases, but their use can also worsen the disease [63]. Targeted biologic and small-molecule therapies for representative chronic inflammatory and autoimmune diseases are given in Table 3.

#### 4.1.1. Infliximab

Infliximab is an IgG1 antibody of high molecular weight that binds both forms of TNF-α, thereby reducing the inflammatory response by modulating cytokine signalling, endothelial adhesion, and immune cell migration, as well as inducing apoptosis of TNF-α–producing cells [77]. Infliximab has been used to treat inflammatory and non-inflammatory diseases: RA, ankylosing spondylitis, active psoriatic arthritis, psoriasis, CD, chronic heart failure, COPD, and selected non-autoimmune inflammatory conditions [78]. Two randomised Norwegian studies, NOR-DRUM A and B, investigated the efficacy of a proactive approach to therapeutic drug monitoring (TDM) during infliximab therapy. NOR-DRUM A focused on achieving remission during induction, while NOR-DRUM B evaluated sustaining remission during long-term treatment. The study included patients with RA, spondyloarthritis, UC, CD, and psoriasis on infliximab for 30 weeks to 3 years. TDM was more effective than standard therapy in maintaining disease control without increasing drug use, but further research is needed to assess its cost-effectiveness and impact on long-term outcomes [63,79]. Due to the rising costs of biological therapies, non-medical switching between infliximab and its biosimilar, CT-P13, is becoming increasingly relevant as a strategy to reduce costs and improve access to treatment for inflammatory diseases [80]. Although existing research, including randomised and observational studies, suggests that this switching does not significantly impact clinical outcomes, the quality of the available data is limited, and the long-term safety and efficacy remain open questions.

Additionally, multidirectional therapy switches in practice have not been sufficiently studied [81,82]. Currently available data on the use of infliximab and other biological drugs in the treatment of inflammatory diseases are limited, so further research should include disease activity, disease outcomes, and long-term practice on the safety and effectiveness of these therapies. At the same time, unresolved medical, ethical, and financial consequences of non-medical switching may pose serious challenges to healthcare systems worldwide in the near future. These issues highlight the need for continued evaluation of biologic treatment strategies, particularly regarding long-term safety, effectiveness, cost, and access.

#### 4.1.2. Adalimumab

Adalimumab is the first human antibody approved by the FDA in 2002 for the treatment of moderate to severe RA, as monotherapy or in combination with other medications. Adalimumab, sold under the name Humira^®^ (AbbVie, Chicago, IL, USA), became the best-selling antibody drug, with global sales exceeding 10 billion USD in 2013 [83]. The most common adverse effects include reactivation of latent infections, a three times higher lymphoma risk, anaphylaxis, CNS disorders, liver toxicity and heart failure. Adalimumab is recommended for CD patients intolerant to infliximab, but not for TNF-blocker-resistant UC [84]. Combination therapy with vedolizumab, adalimumab, and methotrexate has shown potential benefit in selected patients with Crohn’s disease, although current evidence remains insufficient to confirm clear superiority over adalimumab monotherapy [85]. In the SELECT-COMPARE study, upadacitinib demonstrated superior long-term efficacy, with a higher proportion of patients remaining on therapy, while adalimumab had a more favourable safety profile. Both therapies had similar risks for serious adverse events, including MACE, VTE, and malignancies, confirming upadacitinib’s superior efficacy and adalimumab’s continued relevance and safety for RA treatment [86].

#### 4.1.3. Etanercept

Etanercept is the biological drug that was originally approved in 1998 for the treatment of psoriatic arthritis, and later also for RA and juvenile RA [87]. Compared with other TNF-α inhibitors, etanercept had a higher drug retention rate, fewer side effects, and a lower risk of treatment discontinuation in first-line biotherapy for RA, compared with adalimumab and infliximab, and, in second-line biotherapy, it was superior to adalimumab [88].

### 4.2. IL-6 and IL-1 Inhibitors

#### 4.2.1. Tocilizumab

Tocilizumab is a humanised monoclonal antibody that targets the IL-6 receptor and is indicated for the treatment of moderately to severely active RA, Giant Cell Arteritis (GCA), and Systemic Sclerosis-Associated Interstitial Lung Disease (SSc-ILD) in adult patients. In patients aged 2 years and older, it is indicated for Polyarticular Juvenile Idiopathic Arthritis (PJIA), Systemic Juvenile Idiopathic Arthritis (SJIA), and Cytokine Release Syndrome (CRS). Hospitalised adult patients with COVID-19 who are receiving systemic corticosteroids can also be treated with tocilizumab [89,90]. Tocilizumab is an injectable drug with long-term efficacy and safety that can help reduce cardiovascular risk in RA by lowering CRP, IL-6, and cholesterol [91].

Tocilizumab’s humanised structure reduces its immunogenicity compared to murine and chimeric antibodies, making it safer for long-term use with fewer immune reactions. Its immunogenic profile is a key advantage for extended treatments. In systemic sclerosis and ILD, tocilizumab preserved lung function, with tocilizumab-treated patients showing a significantly smaller decline in FVC (352.6 mL) compared to the placebo group (2197.2 mL) over 48 weeks [92]. A recent randomised study examined the impact of tocilizumab and rituximab in patients with RA and found a significant association between treatment effectiveness and synovial histological phenotype. Patients with a diffuse myeloid phenotype showed a notably improved response to tocilizumab (81%) compared with rituximab (35%). Conversely, a higher number of CD8 T cells was correlated with a more favourable response to rituximab, whereas macrophages, monocytes, and myeloid dendritic cells played a key role in the response to tocilizumab. Molecular analyses identified that multidrug-resistant patients exhibited a fibroblast and stromal gene signature, suggesting that new therapeutic approaches could target these cellular populations [93].

#### 4.2.2. Anakinra

Anakinra is an interleukin-1 receptor antagonist approved for the treatment of moderately to severely active RA, cryopyrin-associated periodic syndromes (CAPS), interleukin-1 receptor antagonist deficiency (DIRA), and, in emergency cases, COVID-19-associated pneumonia [94,95]. The EMA has also approved the use of this drug in Still’s disease and familial Mediterranean fever [96]. A recent study [97] demonstrated that anakinra at a dose of 300 mg/day led to clinical improvement, improved quality of life, and reduced inflammatory markers in patients with refractory pustular psoriasis. The study demonstrated that higher doses and longer treatment were crucial for achieving a better response compared to lower doses [98].

### 4.3. JAK-STAT Pathway Inhibitors

**Figure 2:**
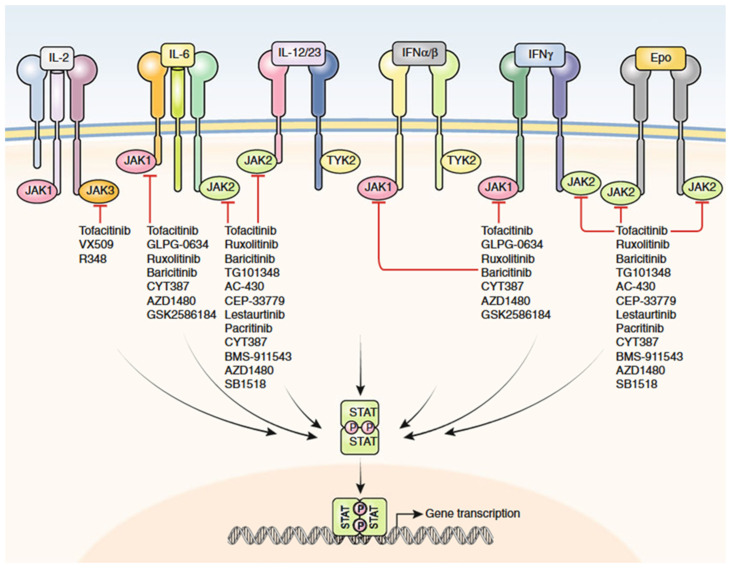
Schematic overview of the JAK-STAT signalling pathway and the site of action of JAK inhibitors. When cytokines bind to their receptors, they activate JAK kinases, usually in pairs. This leads to the activation of STAT proteins, which then move to the nucleus and regulate inflammatory genes. JAK inhibitors block this pathway, but they differ in their selectivity. Tofacitinib does not act equally on JAK1, JAK2, and JAK3. It mainly inhibits JAK1 and JAK3, while its effect on JAK2 may depend on the dose and cellular context. Abbreviations: JAK, Janus kinase; STAT, signal transducer and activator of transcription [101]. Picture is licensed under [CC BY 4.0].

The Janus kinase family includes four members: JAK1, JAK2, JAK3, and tyrosine kinase 2 (TYK2). These kinases usually act in pairs to exert their functions. Some drugs are approved to target JAK family members and primarily target JAK1 or JAK3. The drugs may act directly by altering haematological and biochemical parameters, including haemoglobin, neutrophils, lymphocytes, cholesterol, liver enzymes, and creatine phosphokinase. JAK inhibitors function by preventing the phosphorylation and subsequent activation of specific JAK proteins, as illustrated in Figure 2 [99,100].

#### 4.3.1. Tofacitinib

Tofacitinib is a DMARD and Janus kinase inhibitor that suppresses the immune system and is used in inflammatory diseases in which TNF blockers cannot be tolerated or have not shown improvement. It predominantly inhibits JAK1 and JAK3, with functional effects on JAK2 depending on dose and cellular context. It has been used since 2012 in the treatment of adults with moderately to severely active RA, active psoriatic arthritis, ankylosing spondylitis (AS), UC, and in people older than 2 years in the treatment of polyarticular course juvenile arthritis [102,103,104,105]. In the indicated doses of 5–10 mg twice daily, tofacitinib can lead to lymphoma, skin tumours, cardiovascular consequences and severe infections. Despite an increased risk of herpes zoster and malignancies, tofacitinib has shown greater efficacy than placebo across age groups. Patients treated with tofacitinib achieved endoscopic and clinical remission, along with improved clinical response [106]. In patients with AS, tofacitinib has also been associated with improvements in pain, fatigue, quality of life, and work productivity [107]. When compared to TNF inhibitors in the treatment of RA, tofacitinib carries a higher risk of major adverse cardiovascular events (MACE) and cancer, with incidence rates of 3.4% for MACE and 4.2% for malignancies, compared to 2.5% (MACE) and 2.9% in the TNF inhibitor group [108].

#### 4.3.2. Baricitinib

Baricitinib is an oral selective JAK inhibitor that primarily targets JAK1 and JAK2, unlike tofacitinib, which also inhibits JAK3. It is used in the treatment of SLE, RA, atopic dermatitis, and alopecia areata [109]. Studies involving baricitinib in patients with RA have shown significant efficacy in reducing pain and joint swelling as well as inflammatory markers with a dose of 4 mg compared to placebo and other biologic therapies. Compared with traditional DMARDs, baricitinib shows a higher clinical remission rate [110].

Its use in treating SLE is also supported by findings from the SLE-BRAVE-I study, in which 57% of patients receiving 4 mg of baricitinib achieved a positive response according to the SLE Responder Index (SRI)-4, compared to 46% in the placebo group, but no significant differences were observed in secondary outcomes. The safety profile in SLE patients was consistent with the known risks of baricitinib, including the potential for serious adverse events [111]. Baricitinib has shown an increased risk of infection and thromboembolic events, which was a key safety signal to be considered when prescribing the drug, especially in patients with a history of cardiovascular diseases. Additional reports have compared baricitinib with other JAK inhibitors in selected inflammatory dermatological conditions, but these findings remain outside the main disease focus of this review [112,113].

### 4.4. B-Cell-Targeted Therapies

Therapies that selectively target B lymphocytes, such as rituximab and belimumab, represent a distinct group of biologic agents designed to modulate immune responses in autoimmune conditions, including RA and SLE [114].

Rituximab, a monoclonal antibody targeting the CD20 antigen on B cells, induces B-cell depletion and is used in the management of various autoimmune disorders and B-cell–related hematologic malignancies. Studies have demonstrated that rituximab reduces disease activity in RA, including attenuating synovial inflammation and slowing structural joint damage [115]. Belimumab targets B-lymphocyte stimulator (BLyS), a key survival and activation factor for B cells. It is approved for the treatment of SLE and active lupus nephritis (LN), and primary Sjögren’s syndrome (pSS) in Europe [116]. The combination of rituximab with belimumab in patients with pSS has been superior in terms of safety and efficacy compared to monotherapy. A reduction in disease activity was evident as early as week 12 in patients receiving combination therapy. By week 68, the mean EULAR Sjögren’s Syndrome Disease Activity Index (ESSDAI) was significantly lower in the combination group (5.0 ± 1.27) compared to the placebo group (8.6 ± 1.57). These findings suggest a synergistic effect of belimumab and rituximab in the treatment of pSS, resulting in greater B-cell depletion in salivary glands and improved clinical outcomes compared with either treatment alone [117]. In another study, SLE was investigated; no significant difference in efficacy was observed between combination therapy and monotherapy. While there were improvements in disease markers, such as anti-dsDNA antibodies and B cells, these results were not strong enough to support the use of the combination therapy over monotherapy with belimumab. This difference may be due to distinct pathophysiology in SLE and pSS, highlighting the need for further research to optimise therapies targeting B-cell dysfunction in autoimmune diseases [118]. In contrast, belimumab did not show superior efficacy in a meta-analysis of targeted therapies for generalised myasthenia gravis, suggesting that the benefit of B-cell-targeted therapy may differ across autoimmune conditions [119].

### 4.5. T-Cell Modulators

T-cell modulators, such as abatacept and alemtuzumab, exert their effects by regulating T cells and thus attenuate autoimmune responses [120]. Abatacept is a biological DMARD recommended for the treatment of RA, acting by specifically modulating signals necessary for T-cell activation. It is a fusion protein of CTLA4 and immunoglobulin G1 that inhibits CD28-mediated co-stimulation of T cells by binding to CD80 or CD86, thereby reducing persistent T-cell activity. It is effective in treating RA either as monotherapy or in combination with traditional DMARDs [121].

In the treatment of pSS, abatacept has demonstrated limited clinical efficacy. In the ESSDAI study, no statistically significant difference in the primary outcome was observed between the abatacept and placebo groups after 24 weeks of treatment. Although some secondary outcomes suggested potential therapeutic benefits, their interpretation is limited due to statistical constraints and a possible placebo effect. These findings highlight the substantial limitations of current response criteria in pSS and underscore the need to develop more precise evaluation methods, including advanced histological analyses. Further research is needed better to understand the pharmacodynamics of abatacept in this patient population, as existing treatment regimens may not be adequately tailored to the disease’s unique characteristics [122,123]. Although abatacept did not show significant efficacy in pSS, findings from recent studies involving individuals at high risk of developing RA indicate that this agent may influence early disease mechanisms and slow progression. The APIPPRA trial showed that 12 months of abatacept treatment reduced RA progression and improved clinical and imaging outcomes compared with placebo, although the effects were not fully sustained after treatment cessation [124]. Similarly, the ARIAA trial demonstrated that a 6-month course of abatacept significantly reduced subclinical MRI-detected inflammatory changes and delayed disease onset, with persistent benefits observed even after a 12-month drug-free follow-up. These results underscore abatacept’s promise in delaying onset and modifying the early course of autoimmune diseases, although the variability in treatment response across different conditions raises important questions regarding disease-specific mechanisms, optimal treatment duration, and long-term efficacy [125]. These findings support further evaluation of abatacept in early autoimmune disease settings. Alemtuzumab, a potent biologic agent used in the treatment of MS and selected autoimmune conditions, exerts its therapeutic effect by depleting mature lymphocytes. By binding to CD52, expressed on the surface of both T and B cells, it disrupts immune cell populations that drive inflammation. Its efficacy in reducing disease activity, particularly in MS, is well documented; however, treatment requires rigorous monitoring due to a heightened risk of infusion-related reactions and opportunistic infections. The profound and long-lasting immune modulation offered by alemtuzumab makes it a valuable but high-stakes option in immune-mediated disease management [126].

## 5. Novel Small-Molecule Inhibitors in the Treatment of Autoimmune and Chronic Inflammatory Diseases

An overview of small molecules used in the treatment of autoimmune and inflammatory diseases is provided in Table 4.

### 5.1. Targeting Intracellular Pathways: Kinase Inhibitors as Precision Therapy

The previously mentioned kinase inhibitors enable precise, targeted therapy for various diseases by modulating immune signalling pathways. The efficacy of these drugs is also reflected in improved outcomes compared with standard monotherapy in selected settings [134,135].

JAK inhibitors play a crucial role in signal transduction from cytokine receptors; upon activation, they phosphorylate and dimerise the STAT protein, which, upon entering the nucleus, modifies gene expression [99]. Baricitinib, a JAK inhibitor, has become a cornerstone in the treatment of autoimmune conditions due to its ability to modulate immune responses, making it a versatile tool for managing a broad range of diseases [136]. Similarly, tofacitinib has shown a promising role in the treatment of psoriatic arthritis compared to adalimumab and otilimumab [137,138,139]. Other JAK inhibitors have also shown therapeutic benefit in selected non-autoimmune conditions, although these indications are outside the main disease focus of this review [140]. Greater drug selectivity also leads to reduced side effects, as seen with selective JAK1 inhibitors such as upadacitinib [141].

In autoimmune diseases, the use of tyrosine kinases is limited by adverse effects, such as cardiovascular complications and skin toxicity, although they are highly effective in treating B-cell malignancies. The selectivity of these drugs, such as ibrutinib and acalabrutinib, has enabled immunotherapy advances and reduced adverse drug effects, except for atrial fibrillation, which remains challenging [142]. Ibrutinib is associated with a 1.5- to 4.1-fold higher incidence of adverse events and a 2- to 2.8-fold higher risk of cardiovascular and bleeding problems compared to acalabrutinib [143]. In addition, these drugs increase the risk of bleeding and severe bleeding [127]. The new oral inhibitor of spleen tyrosine kinase (SYK), sovleplenib, is used in the treatment of chronic primary immune thrombocytopenia (ITP) by reducing platelet destruction and increasing platelet production. Compared with placebo, it provides a durable response in 48% of patients with chronic primary thrombocytopenia. It has the advantage of showing few to moderate adverse events [144].

The therapeutic potential of kinase inhibitors is great, and there are high expectations for these drugs in addressing complex diseases. However, their future role will depend on achieving a balance between efficacy, selectivity, safety, and disease-specific application.

### 5.2. Sphingosine-1-Phosphate Receptor Modulators

S1P signalling pathways play a crucial role in immune cell trafficking, vascular permeability, and neurogenesis. S1P receptor modulators are orally administered small molecules that can act as either antagonists or agonists, depending on the receptor subtype and target tissue. The most important receptor, S1PR1, regulates processes like angiogenesis, immune cell trafficking, and vascular tone. The first approved S1PR modulator, fingolimod, is used to treat multiple sclerosis (MS) [129]. However, its non-selective activity across multiple S1PR subtypes may cause adverse effects, including bradycardia, hypertension, and prolonged lymphocyte depletion. To address these limitations, newer modulators such as ozanimod, siponimod, ponesimod, and etrasimod selectively target specific S1PR subtypes, reducing side effects and shortening their half-lives. These drugs are already approved for MS, with ozanimod also licensed for UC [130,131].

A recent trial on Icanbelimod (CBP-307), a next-generation modulator targeting S1PR1, showed promising results in reducing lymphocyte counts in healthy individuals, with a dose-dependent response and quick recovery of lymphocyte levels. This drug was well-tolerated with mild side effects, further supporting the therapeutic potential of selectively targeting specific S1PR subtypes in autoimmune diseases [145]. Cenerimod, which also targets S1PR1, has shown potential in treating SLE, with the 4.0 mg dose demonstrating significant improvement, although the primary endpoint was not met. With good tolerability over a 12-month period and no treatment- or dose-related adverse effects reported so far, this drug, along with many other future S1P receptor modulators, may support broader, yet still disease-specific, applications in selected immune-mediated diseases [146].

### 5.3. PDE4 Inhibitors

Phosphodiesterase 4 (PDE4) is an intracellular enzyme that regulates inflammation and maintains epithelial integrity by degrading cyclic adenosine monophosphate (cAMP). Due to its pivotal function in inflammatory processes, this enzyme is one of the primary therapeutic targets for the treatment of neurological, dermatological and pulmonary diseases [147]. Several PDE4 inhibitors (apremilast, crisaborole, roflumilast) have been approved for the treatment of inflammatory diseases, but despite clinical efficacy, these agents are frequently associated with adverse effects such as nausea and gastrointestinal discomfort. Ongoing advancements in drug development have focused on refining PDE4 inhibitors to enhance their therapeutic potential while mitigating undesirable side effects [133]. Apremilast received its first paediatric approval in 2024 for the treatment of moderate to severe plaque psoriasis in children aged six years and older who are candidates for systemic therapy [132].

## 6. Role of Cell-Based and Gene Therapies

Cell-based and gene therapies are among the novel therapeutic approaches. The main characteristics summarised are given in Table 5. However, these therapies are still in very limited and controlled use. Mostly, it requires a clinical environment and specifically educated staff, apart from high costs. These are the main limitations of this therapeutic approach.

### 6.1. Stem Cell Therapy

**Figure 3:**
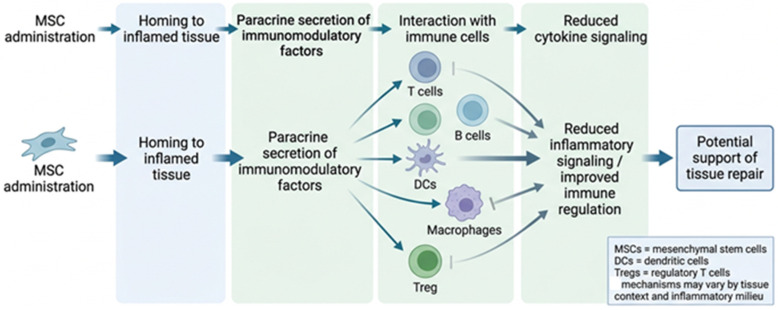
Stem Cell-Based Immunomodulation. Created in Canva (https://www.canva.com). Once injected, MSCs migrate to sites of inflammation, where they release paracrine factors that act on immune cells, including T cells, B cells, dendritic cells, macrophages, and regulatory T cells. This may limit inflammation, but the final outcome depends on the circumstances. Figure was created in Canva by the authors.

Stem cells have a significant capacity for self-replication and for transforming into different cell types, making them crucial in regenerative medicine and therapies for autoimmune diseases. They can be categorised into various types (totipotent, pluripotent, multipotent, oligopotent, and unipotent) [148]. Embryonic stem cells (ESCs) are pluripotent and can differentiate into all three primary germ layers, whereas adult stem cells, usually multipotent, are involved in the regeneration of specific tissues. Foetal stem cells also demonstrate pluripotency, allowing them to give rise to various tissues present during prenatal development [149]. Among stem cells, mesenchymal stem cells (MSCs) and induced pluripotent stem cells (iPSCs) show promising potential for treating autoimmune diseases, as shown in Figure 3. MSCs migrate to sites of inflammation, guided by a variety of biochemical signals, mostly by proinflammatory cytokines such as TNFα, IL1, and IFNγ [150].

Moderate levels of these cytokines promote MSC migration, whereas excessive levels can impair their function in autoimmune conditions [151]. MSCs modulate the function of immune cells, including T and B lymphocytes, dendritic cells (DCs), and NK cells. By activating and proliferating T and B cells, MSCs also simultaneously activate Tregs, which is crucial for maintaining immune balance and control in autoimmune diseases such as RA, SLE, and MS. MSCs affect DCs by reducing their differentiation and migration, thereby preventing antigen presentation. They also regulate B-cell activity through factors such as the IL-1 receptor antagonist and, indirectly, through T-helper cells [152,153]. In a recent meta-analysis [154], stem cell therapy (SCT), particularly in combination with autologous hematopoietic stem cell transplantation (AHSCT), can improve clinical outcomes in MS patients, including reductions in EDSS scores and MRI lesion volume.

Although no significant improvements in walking ability or upper extremity function were observed, patients who received SCT had a lower incidence of infections than the control group. This effect may be related to MSCs’ immunomodulatory and anti-inflammatory properties, even without intensive immunosuppression. Another meta-analysis [155] on MSC therapy in RA patients showed no differences in adverse events between the MSC and control groups, but no sustained benefit was observed after 12 months without continuous treatment. In another SLE study [156], Dexlip-MSCs (MSCs integrated with dexamethasone) showed efficacy in inhibiting T-cell proliferation and reducing proinflammatory cytokine release, suggesting a promising therapeutic approach for managing disease progression. Stem cell therapies, particularly MSCs, have significant potential for treating autoimmune diseases, but ethical considerations are crucial. Autologous stem cell therapy, which utilises MSCs derived from the patient’s own tissue, reduces the likelihood of immune rejection and adheres to ethical standards for cell harvesting [157].

### 6.2. CAR-T and Treg Therapy

Although originally developed for cancer treatment, CAR-T cell therapy has shown promise in treating autoimmune diseases [158]. In its structure, CAR has four domains: the first ligand-binding domain, which enables antigen recognition; the spacer domain; the transmembrane domain; and the cytoplasmic domain [159]. With the development of new generations of CAR-T cells, the ligand-binding domain has been most affected, and in the second generation, this domain included the costimulatory domains CD28 and 4-1BB (CD137). In the third generation, multiple costimulatory domains were added, allowing these cells to persist long-term in the body and improving function [160].

CD19, which is expressed on both normal and malignant B cells, is the most extensively studied target in CAR-T therapy. By focusing on CD19, the FDA approved CAR-T treatment due to its success in eliminating malignant B cells [161]. This therapeutic success has paved the way for the potential use of B-cell-depleting strategies in the management of autoimmune diseases. Furthermore, emerging approaches such as targeted elimination of autoantigens, dual-antigen targeting, and the engineering of regulatory T cells are under active investigation and will be further elaborated [162,163,164]. A case series of 15 patients with therapy-resistant autoimmune diseases, including SS, inflammatory myositis (IIM), and SLE, showed that CD19-targeted CAR-T cell therapy resulted in sustained remission in most SLE cases and significant clinical improvement in others, with minimal side effects and a low rate of infections. This demonstrated the therapy’s potential as an alternative to conventional immunosuppressive strategies [165].

CAR-T therapy may offer advantages over conventional therapy because it targets autoreactive immune cells more directly and may modify key disease-driving mechanisms. In contrast, conventional therapy often focuses on controlling downstream inflammation and symptoms. In this way, CAR-T therapy enables long-term remission for patients and represents a promising therapy for autoimmune diseases, although challenges in its clinical application remain [164]. Future research should prioritise the identification of novel antigenic targets beyond CD19, particularly those involved in non–B-cell-mediated autoimmunity. Additionally, the development of dual-specific or regulatable CAR constructs, as well as rigorous long-term safety studies in diverse patient populations, will be crucial to fully establish the therapeutic potential of CAR-T and Treg-based interventions in autoimmune settings.

Regulatory T cells (Tregs) are key effectors of immune tolerance and are known to downregulate autoreactive immune responses. Thus, Tregs are an emerging therapeutic platform for autoimmune disease. In contrast to conventional immunosuppressive approaches, which are non-specific, Tregs are being engineered to induce antigen-specific tolerance. In addition, emerging therapeutic modalities such as engineered Tregs, including CAR-Tregs, are being developed to enhance the specificity, persistence, and suppressive function of Tregs. These therapeutic modalities are promising for controlling autoimmune disease with minimal systemic side effects [26].

### 6.3. CRISPR and Gene Editing

Autoimmune diseases are influenced by various genetic factors, making gene therapy a promising therapeutic strategy. Among advanced gene-editing techniques, CRISPR-Cas9 stands out as highly effective. CRISPR-Cas9 enables precise gene modification by using a synthetic single-guide RNA (sgRNA) that directs the Cas9 nuclease to a specific target sequence. Following the induction of a double-strand break in DNA, gene disruption can occur via non-homologous end joining (NHEJ), whereas precise gene insertion is possible through homologous recombination (HDR), provided an appropriate DNA template is introduced [166,167]. In the study by Yang et al. [168], the MYC and FOXO1 genes were investigated in the context of RA pathogenesis. Their findings showed that CD4+ T cells from RA patients exhibit increased autophagy, with MYC playing a central regulatory role. Additionally, FOXO1 was significantly associated with disease progression. These findings highlight the potential of MYC and FOXO1 as targets for CRISPR-Cas9 therapy, enabling genetic modification that could downregulate CD4+ T cell activation. While these results are promising, their application in humans requires further investigation and clinical studies to assess the real efficacy of CRISPR-Cas9 therapies.

Similarly, Li and colleagues [169] investigated the role of the PTPN2 gene in CD patients. Mutations in the PTPN2 gene, particularly the SNP rs7234029, led to functional changes affecting its gene expression. Using CRISPR-Cas9 to knock out PTPN2, the researchers observed increased phosphorylation of STAT3 and Erk1/2, as well as increased cell proliferation, indicating that these genetic variants play a role in CD’s pathogenesis. While these findings are highly promising, further studies are required to assess the efficacy of CRISPR-Cas9 in human models. The majority of current research uses cellular models, and their application in humans is still in its early stages. CRISPR-Cas9 faces several limitations, including unintended off-target effects, difficulties with efficient delivery, and significant ethical concerns, particularly when applied to human germline editing [170].

## 7. Advances in Microbiome and Gut–Immune Axis Modulation

**Figure 4:**
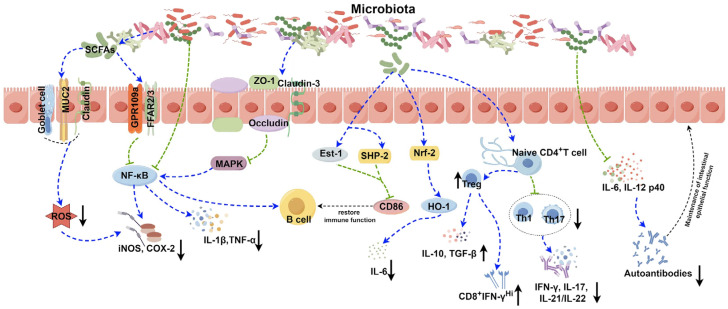
Microbiome-Based Therapies and Their Mechanisms [175]. The figure is licensed under [CC BY 4.0].

The gut microbiome is an adaptable, complex system, shaped by the environment, dietary habits, and host immune status. The composition of the gut microbiome and its functional capacity change throughout life, influencing immune regulation and the pathogenesis of autoimmune diseases. Recent advances have highlighted the gut microbiome’s ability to modulate inflammation by influencing immune cell activation, autoantibody generation, and disease progression (Figure 4) [171]. Beyond its role in maintaining local microbial balance, the intestinal niche serves as a critical immunological interface. Gut-associated lymphoid tissue (GALT), in conjunction with mucosa-associated lymphoid tissue (MALT), houses innate immune cells that initiate early pathogen recognition and present antigens to the adaptive immune system. Gut-resident macrophages, influenced by microbial cues, not only defend against pathogens but also maintain epithelial homeostasis. Dysbiosis-induced dysfunction of this axis has been linked to chronic inflammation and autoimmune pathogenesis, including SLE and RA [172,173]. The gut microbiota directs the differentiation of naïve CD4+ T cells into either proinflammatory Th17 cells or Tregs, tipping the immunological balance. Th17 cells release IL-17 and IL-22, contributing to local immune defence, while Tregs preserve tolerance and prevent excessive immune activation. Microbial antigens, such as Ro60, have been implicated in activating autoreactive T and B cells in SLE, underscoring the pathogenic potential of microbiome-immune dysregulation [174].

Following the discovery of the microbiome’s therapeutic potential in treating autoimmune diseases, there has been increased interest in the role and application of prebiotics, probiotics, and postbiotics in managing these conditions. Particular attention has been given to faecal microbiota transplantation (FMT), which was initially used to treat Clostridioides difficile infections [176]. When functional microbiota from the stool of a healthy donor is transferred to a patient to modify their microbiome, this is an FMT procedure. The goal of the procedure is to rebalance the patient’s microbiome and strengthen their intestinal barrier, thereby regulating immune cell activity [177,178].

A recent systematic review [179] highlighted notable differences in gut microbial profiles between individuals with IBD and healthy subjects. In patients diagnosed with CD, increased abundance of *Escherichia coli*, *Veillonella*, and *Actinomyces* was observed, accompanied by reduced abundance of beneficial taxa such as *Faecalibacterium prausnitzii*, Coriobacteriaceae, and Christensenellaceae. Similarly, altered microbial signatures have been reported in MS, with elevated levels of *Akkermansia* and *Methanobrevibacter*, and a noticeable decrease in *Butyricimonas*, suggesting a potential link between microbial composition and autoimmune pathology. These findings support the relevance of microbiome-targeted interventions in representative chronic inflammatory and autoimmune diseases. Fermented foods in this study were associated with increased microbiome diversity and reduced inflammation markers. This suggests that fermented foods may reduce inflammation by reshaping the microbiota [179].

In contrast, while a diet rich in fibre did not result in a rapid increase in microbiota diversity, it was associated with shifts in microbial composition that enhanced the synthesis of short-chain fatty acids (SCFAs), which are recognised for their anti-inflammatory properties. Although the increase in microbiome diversity was slower, the fibre-rich diet still showed promise in reducing inflammation through these metabolic changes. While both high-fibre and fermented-food diets influence inflammation and the microbiome, combining these dietary components could have a synergistic effect. Further studies are needed to clarify how these interventions can be incorporated into personalised approaches to reduce chronic inflammation and prevent related diseases [177]. Table 6 gives an overview of microbiome-based strategies for the treatment and modulation of autoimmune and chronic inflammatory conditions.

## 8. Nanotechnology and Drug Delivery Innovations

### 8.1. Liposomal Drug Formulations for Targeted Therapy

**Figure 5:**
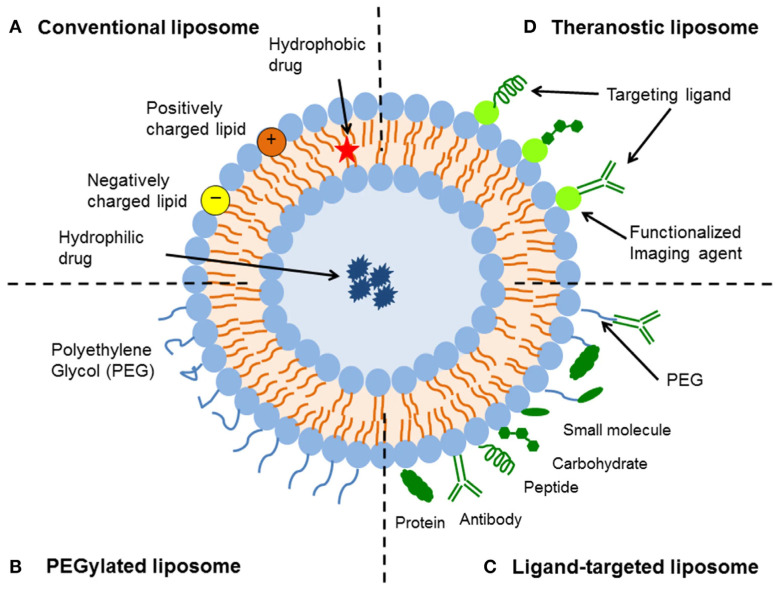
Liposome Drug Delivery for Autoimmune Diseases [187]. Figure is licenced under [CC BY 4.0].

Liposomes are drug delivery systems that improve bioavailability, minimise toxicity, and enable targeted treatment by encapsulating both hydrophilic and hydrophobic drugs, thereby enhancing pharmacokinetics and controlled release (Figure 5). Methotrexate (MTX), widely used for autoimmune and inflammatory diseases, has limitations such as toxicity and low retention. Liposomal encapsulation improves drug stability, reducing systemic toxicity while maintaining efficacy [184,185]. However, Guimarães et al. [186] demonstrated that optimising encapsulation parameters significantly enhances MTX retention, preventing rapid leakage and loss. This refinement offers a model for improving liposomal formulations of highly charged drugs. Table 7 gives an overview of nanotechnological solutions, nanocarriers and nanodrugs used for immune related diseases.

This advancement in liposomal drug delivery extends beyond MTX, opening new possibilities for enhancing the effectiveness and bioavailability of other highly charged drugs. In recent years, nanotechnology-based drug delivery platforms, such as liposomes, have been increasingly explored for their potential to improve drug absorption, distribution, and controlled release. Their structural properties allow them to transport both hydrophobic and hydrophilic molecules efficiently, further expanding their applications in modern pharmacotherapy [194]. Liposomal dexamethasone formulations have also been explored in other disease settings, showing the potential of this platform to increase drug exposure while reducing systemic toxicity [195]. Although these data are encouraging, their relevance to autoimmune and chronic inflammatory diseases still requires further focused evaluation.

### 8.2. Nanoparticles and mRNA-Based Therapies

Engineering immunomodulatory nanoparticles gained attention in recent years due to their ability to interact with immune cells. These nanoparticles can be selectively internalised by APCs, particularly DCs, and influence the activation and regulation of immune responses [196].

Dendritic cells are central to immune surveillance and modulation, primarily through their role in antigen presentation. In the context of autoimmune diseases, their dysregulation contributes to abnormal T-cell activation and breakdown of self-tolerance. The ability of nanoparticles to target DCs offers an opportunity to regulate immune responses by promoting Treg induction and shifting the balance from a proinflammatory to a tolerogenic state. Nanoparticles can be designed to enhance antigen presentation in a controlled manner, ensuring that autoantigens are processed in a tolerogenic rather than an immunogenic way. Nanoparticle selectivity can be achieved by surface conjugation with ligands that target specific receptors on dendritic cells. In addition, nanoparticles can promote an anti-inflammatory environment by carrying tolerogenic cytokines or immunosuppressive agents (e.g., dexamethasone) [197]. One such example is the PDMAEMA-PLGA nanoparticle carrying dexamethasone, which switches DC from an immunogenic to a tolerogenic state, thereby alleviating symptoms in a model of SLE [198].

Nanoparticles can also modulate T cell function, and engineered nanoparticles that mimic APCs can be used to promote antigen-specific tolerance by expanding Treg cells while simultaneously suppressing the activation of effector T cells [199]. A further step in the use of nanoparticles is their delivery of immune checkpoint modulators to autoreactive T cells. A recent advance is the use of biodegradable nanoparticles loaded with small molecules that inhibit the production of proinflammatory cytokines [200]. Beyond their effects on adaptive immunity, nanoparticles can also alter the activity of macrophages and NK cells involved in inflammation in autoimmune diseases [201].

Challenges in using nanoparticle-based therapies include optimising nanoparticle design to ensure targeted delivery, reducing off-target effects, and increasing biocompatibility. Future research should focus on translating these findings into clinical applications and on improving nanoparticle formulations to achieve accurate immune regulation with minimal side effects. Young et al. [202] demonstrated that modified selenium nanoparticles (M-SeNPs) combined with chitosan/sodium alginate (CS/SA) hydrogels can target the colon and reduce inflammation. This approach inhibits the NF-κB pathway, increases glutathione peroxidase (GPX) expression, and modulates the immune response of enterocyte cells (IECs). A promising approach to treating IBD involves modifying the mannose on nanoparticles to enable specific binding to receptors on IECs. To assess patients’ response to TNF-α therapy in UC, a model focusing on mRNA has been developed. Based on this model, patients are better stratified into those who respond to treatment and those who do not. One factor that also affects treatment response is mitochondrial dysfunction. This mRNA-based model can target mitochondrial pathways, especially in patients who do not respond to conventional therapies [203].

## 9. Artificial Intelligence and Machine Learning in Drug Discovery

Advances in artificial intelligence and machine learning have enabled the analysis of large amounts of data on repurposed drugs for the treatment of autoimmune diseases. Molecular profile analysis, when integrated with clinical data, reveals common signatures in diseases such as SLE, pSS, and RA [204,205]. This has led to the identification of disease factors and potential drug repurposing targets, offering new therapeutic options for patients with autoimmune disease [206]. A recent study explored AI’s role in predicting autoimmune diseases, focusing on T-cell receptors (TCRs). Two deep learning models, AutoY (using a CNN) and LSTMY (using a bidirectional LSTM with attention mechanisms), achieved high accuracy, with AUC values of 0.99 for type 1 diabetes and multiple sclerosis, demonstrating AI’s potential to improve the diagnosis and treatment of autoimmune diseases [207]. Examples of repurposed or newly developed identities are given in Table 8.

Given the variability and high comorbidity rates of autoimmune diseases, personalised medicine is essential, as it focuses on the molecular mechanisms of these diseases rather than treating their consequences. Healthcare generates various types of clinical data, and artificial intelligence and machine learning identify patterns within large datasets. This approach enables risk assessment, disease management, monitoring, and outcome prediction, particularly for autoimmune diseases [208].

Machine learning has shown promise in diagnosing and treating autoimmune diseases, particularly SLE, by identifying biomarkers, guiding drug repurposing, and improving diagnostic accuracy. Predictive models help forecast disease risk, outcomes, and treatment responses, enabling personalised therapy. Research on SNP and GWAS data helps develop personalised therapies by identifying genetic variants and improving patient stratification. Combining genetic findings with clinical data refines treatment options for autoimmune diseases such as RA and IBD, with techniques such as random forests and support vector machines enhancing disease management [209,210]. A recent study [211] used proteomics and RNA sequencing to identify biomarkers that differentiate active from inactive SLE, thereby contributing to more targeted and personalised therapies.

Artificial intelligence, by analysing multi-omics data and integrating them with clinical information, has helped improve our understanding of the molecular signatures associated with autoimmune diseases and may support more precise targeting of underlying disease mechanisms [212]. Similarly, AI can virtually create a digital twin of a patient based on the patient’s medical data. Using these digital examples, healthcare professionals can simulate the progression of the disease and monitor treatment response [213]. While AI and ML technologies offer considerable promise for diagnosing and managing autoimmune diseases, several challenges still prevent their full implementation. One major issue is the inconsistency in reporting ML models, with many studies failing to adhere to clear reporting standards.

Additionally, challenges such as data quality, model interpretability, and validation across diverse patient populations remain significant obstacles. While AI approaches are widely applicable in drug discovery and clinical research, they rely heavily on large, reliable datasets, which are often limited in autoimmune disease research. Gathering diverse and representative datasets that accurately reflect the population is critical for accurate predictions, but issues such as varying annotation standards, data noise, and access limitations continue to pose significant challenges [214,215]. Applying AI in drug development and the management of autoimmune diseases raises ethical considerations, including protecting patient data, ensuring algorithmic transparency, and ensuring equitable outcomes. AI models must be developed and applied responsibly to ensure inclusivity and avoid biases that could exacerbate healthcare disparities, particularly in conditions with high variability in clinical presentation and patient demographics [216].

Addressing these challenges requires a multifaceted approach. Enhanced collaboration between researchers and industry through data sharing can improve sample diversity. Standardised data processing and labelling are essential for generating reliable and comparable outcomes [209]. As AI and ML technologies continue to evolve, addressing issues of model interpretability and validation across diverse populations will enable broader application in immunology, thereby supporting more specific and effective drug discovery strategies for autoimmune diseases [217].

There are also challenges to applying pharmacogenomic approaches in clinical practice, including the complexity of genetic variability, limited clinical evidence, and the interactions among genetic, environmental, and lifestyle factors that affect drug metabolism and response [218]. High costs and patient-related factors, such as acceptance and adherence, limit clinical adoption of pharmacogenomic testing [209]. However, AI-based methods can still identify novel drug combinations and dosage regimens, offering significant promise in applying already established treatments to new indications. These methods could be priority candidates for further research and validation, ultimately providing more personalised and effective therapies for autoimmune diseases.

In conclusion, although the path to integrating AI and ML into the care of autoimmune diseases is complex, their thoughtful and responsible implementation has the potential to transform clinical decision-making and patient trajectories. As methodologies mature and data systems become more refined, AI and ML may help support a more nuanced, precise, and clinically useful approach to immune-mediated disorders, while still requiring careful validation and responsible use. Future research should focus on standardising data annotation practices and enhancing the transparency of machine learning algorithms to improve reproducibility and clinical trust. Additionally, efforts should prioritise the development of integrative, multimodal AI platforms capable of translating complex omics, imaging, and clinical data into actionable therapeutic insights.

## 10. Conclusions

Chronic inflammatory diseases (CIDs) and autoimmune diseases have been on the rise in recent years, especially in women. Conventional therapies focus more on symptom control than on the mechanisms of inflammation. Additionally, many patients fail to achieve satisfactory responses and, through long-term immunosuppression, further impair the human immune system, making it more susceptible to infections and malignancies. The lack of standardised dosing and treatment duration across many therapies further complicates care, underscoring the need for well-designed studies to determine optimal regimens. Key findings highlight a clear need for novel therapies, including nanotechnology, monoclonal antibodies, therapeutic vaccines, and the use of artificial intelligence and machine learning.

The heterogeneity of these diseases clearly underscores the need for personalised medicine and an individualised approach to each patient. At the same time, the comparative review of representative chronic inflammatory and autoimmune diseases in this manuscript suggests that future therapeutic progress will depend not only on innovation but also on disease-specific targeting, careful patient stratification, and long-term safety evaluation. Precision medicine is not only a technological innovation but a commitment to safer and compassionate care. The main limitation of this study lies in the limited number of high-quality, multicenter clinical trials in humans. Future research on novel therapies should focus on the pathophysiology and mechanisms of disease, long-term outcomes, the costs of such treatments, and ethical transparency.

## Figures and Tables

**Table 1:** Representative Chronic Inflammatory Diseases and Autoimmune Diseases Included in This Review.

Disease Category	Disease	Prevalence	Pathophysiology Features	Mainly Affected Organs	References
Autoimmune disease	Rheumatoid arthritis (RA)	500 cases per 100,000 adults, affects women 2–3 times more often than men, with peak incidence in the sixth decade.	Chronic synovial inflammation causes cartilage and bone damage, and immune cell infiltration (T cells, B cells, monocytes). Key molecules: TNF, IL-6, RANKL, prostaglandins, and matrix metalloproteinases.	Joints (hands, feet, wrists, ankles, knees, elbows, shoulders), skin (rheumatoid nodules), cardiovascular system, lungs (interstitial lung disease), vasculature (rheumatoid vasculitis).	[13,14]
Autoimmune disease	Systemic lupus erythematosus (SLE)	43.7 cases per 100,000	Production of autoantibodies (e.g., anti-dsDNA, anti-Smith) that form immune complexes and cause inflammation in various organs.	Affects skin, joints, kidneys, heart, lungs, CNS, blood and GI tract.	[15,16]
Chronic inflammatory disease	Inflammatory bowel disease (IBD)	59.25 per 100,000	Chronic relapsing intestinal inflammation driven by dysregulated mucosal immune responses, barrier dysfunction, genetic susceptibility, and altered gut microbiota.	Gastrointestinal tract, primarily the small intestine and colon; extraintestinal manifestations may involve the joints, skin, eyes, and hepatobiliary system.	[17]
Autoimmune disease	Multiple sclerosis (MS)	5 to 300 per 100,000	Key immune cells: CD8+ T cells, B cells, plasma cells; cytokines: IFN-γ, TNF-α, IL-17, IL-1, IL-6, IL-10.	Central nervous system (CNS), including the brain, spinal cord, and optic nerves.	[18,19]
Autoimmune disease	Hashimoto’s thyroiditis	0.3–1.5 cases per 1000, more common in women; increases with age	Autoimmune disorder characterised by lymphocyte infiltration of the thyroid, presence of anti-thyroid antibodies (anti-TG, anti-TPO), dysfunction of B and T cells, and involvement of cellular and humoral immunity.	Thyroid gland (primary), cardiovascular system and other organs are affected due to associated hypothyroidism and autoimmune conditions.	[20]
	Psoriasis	Ranges from 0.1% in East Asia to 1.5% in Western Europe; higher rates are seen in high-income regions.	Involves genetic factors, immune system dysregulation, particularly IL-17, IL-23, and TNFα; triggers include stress, infections, drugs, and obesity.	Primarily affects the skin, especially the knees, elbows, scalp, and sometimes palms, soles, and genitalia.	[21]

**Table 2:** Conventional therapies for representative chronic inflammatory and autoimmune diseases.

Therapy Type	Mechanism of Action	Efficacy	Limitations	Examples of Drugs	Ref.
NSAIDs (Nonsteroidal Anti-inflammatory Drugs)	Inhibition of cyclooxygenase enzymes (COX-1 and/or COX-2), reducing prostaglandin synthesis	Effective in reducing pain and inflammation, commonly used in the early stages	gastrointestinal problems, kidney issues, increased risk of cardiovascular diseases	Ibuprofen, diclofenac, naproxen, meloxicam, indomethacin	[42]
Corticosteroids	Suppression of inflammation and immune response through inhibition of proinflammatory cytokines	Rapid effectiveness in reducing acute inflammation and symptoms	Hypertension, susceptibility to infections, bone density loss, weight gain, and diabetes mellitus	Prednisone, methylprednisolone, dexamethasone, hydrocortisone	[43]
DMARDs (Disease-modifying Antirheumatic Drugs)	Modulate immune responses through inhibition of folate-dependent pathways, lymphocyte proliferation, cytokine production, or antigen presentation, depending on the agent	Effective in modifying disease course, especially in autoimmune disorders such as rheumatoid arthritis	Can have serious side effects, including liver toxicity, kidney issues, and hematologic effects	Methotrexate, leflunomide, sulfasalazine, hydroxychloroquine, azathioprine	[44]
Biologics (Biological Agents)	Target specific molecules or cells involved in inflammation, such as TNFα, IL-6, IL-17, IL-23	Effective in chronic autoimmune disorders (RA, psoriasis, CD)	High cost, may cause immune reactions, infections, and malignancies	IL-6 inhibitors (tocilizumab), TNF inhibitors (etanercept, infliximab, adalimumab), IL-17 inhibitors (secukinumab)	[45,46]

**Table 3:** Targeted biologic and small-molecule therapies for representative chronic inflammatory and autoimmune diseases: mechanisms, selectivity, indications, efficacy, and safety considerations.

Therapy Type	Drug/Target Selectivity	Mechanism of Action	Efficacy	Original Product	FDA Approval Year (First-Latest Indication)	EMA Approval Year	Therapeutic Indications	Side Effects	References
TNF-α inhibitors	Etanercept—soluble TNF receptor fusion protein	Neutralise TNF-α and prevent TNF receptor-mediated inflammatory signalling	Highly effective in reducing inflammation and controlling disease activity in representative autoimmune and chronic inflammatory diseases	Enbrel	1998–2016	2000	Rheumatoid arthritis, polyarticular juvenile idiopathic arthritis, psoriatic arthritis, ankylosing spondylitis, plaque psoriasis, paediatric plaque psoriasis	Injection-site reactions; infections, including tuberculosis reactivation; congestive heart failure; cytopenias; demyelinating events; autoimmune phenomena	[64,65,66,67]
	Infliximab—chimeric monoclonal antibody against TNF-α			Remicade	1998–2011	1999	Crohn’s disease, rheumatoid arthritis (in combination with methotrexate), ankylosing spondylitis, psoriatic arthritis, ulcerative colitis, plaque psoriasis, paediatric Crohn’s disease, paediatric ulcerative colitis		
	Adalimumab—fully human monoclonal antibody against TNF-α			Humira	2002–2021	2006	Rheumatoid arthritis, psoriatic arthritis, Crohn’s disease, psoriasis, juvenile idiopathic arthritis, ulcerative colitis, hidradenitis suppurativa, uveitis		
	Certolizumab—PEGylated Fab’ fragment targeting TNF-α			Cimzia	2008–2019	2009	Crohn’s disease, rheumatoid arthritis, psoriatic arthritis, plaque psoriasis, ankylosing spondylitis		
	Golimumab—human monoclonal antibody against TNF-α			Simponi	2009–2013	2009	Ankylosing spondylitis, moderate to severely active rheumatoid arthritis (in combination with methotrexate)		
IL-6 inhibitors	Tocilizumab—humanised monoclonal antibody targeting the IL-6 receptor	Block interleukin-6 (IL-6), a cytokine involved in immune response and inflammation	Effective in reducing disease activity in rheumatoid arthritis, systemic juvenile idiopathic arthritis, and cytokine release syndromes	Actemra/RoActemra	2010–2022	2009	Rheumatoid arthritis, giant cell arteritis, systemic sclerosis-associated interstitial lung disease, polyarticular juvenile idiopathic arthritis, systemic juvenile idiopathic arthritis, cytokine release syndrome, COVID-19	Infection, neutropenia, hyperlipidaemia, thrombocytopenia, abnormal liver enzymes	[66,67,68,69]
	Sarilumab—human monoclonal antibody targeting the IL-6 receptor			Kevzara	2017–2024	2017	Rheumatoid arthritis, polymyalgia rheumatica, polyarticular juvenile idiopathic arthritis		
JAK inhibitors	Tofacitinib—predominantly JAK1/JAK3 inhibitor; functional JAK2 effects may occur depending on dose and cellular context	Inhibit Janus kinase-mediated intracellular signalling involved in inflammatory and immune responses	Effective in treating rheumatoid arthritis, psoriatic arthritis, and ulcerative colitis	Xeljanz	2012–2021	2017	Rheumatoid arthritis, psoriatic arthritis, ankylosing spondylitis, ulcerative colitis, polyarticular course juvenile idiopathic arthritis	Infection, embolism, thrombosis, neoplasms, gastrointestinal perforation events, musculoskeletal and connective tissue disorders	[70,71]
	Baricitinib—primarily JAK1/JAK2 inhibitor			Olumiant	2018–2022	2017	Rheumatoid arthritis, COVID-19, alopecia		
	Upadacitinib—preferential JAK1 inhibitor			Rinvoq	2019–2023	2019	Rheumatoid arthritis, psoriatic arthritis, atopic dermatitis, ulcerative colitis, Crohn’s disease, ankylosing spondylitis, non-radiographic axial spondyloarthritis, polyarticular juvenile idiopathic arthritis		
B-cell therapies	Rituximab—anti-CD20 monoclonal antibody causing B-cell depletion	Target B-cell survival or depletion pathways to reduce autoantibody-driven and B-cell-mediated inflammation	Highly effective in diseases like rheumatoid arthritis and lupus, where B cells play a key role in disease progression	Rituxan/MabThera	1997–2023	1998	Chronic lymphocytic leukaemia, rheumatoid arthritis, non-Hodgkin’s lymphoma, granulomatosis with polyangiitis, microscopic polyangiitis, pemphigus	Infection, neutropenia, hypogammaglobulinemia, hypersensitivity	[66,67,72,73,74]
	Belimumab—monoclonal antibody targeting B-lymphocyte stimulator (BLyS/BAFF)			Benlysta	2011–2024	2011	Lupus erythematosus (patients 5 years and older with standard therapy), lupus nephritis		
T-cell modulators	Abatacept—CTLA4-Ig fusion protein blocking CD80/CD86-CD28 co-stimulation	Modulate T-cell activation or deplete lymphocyte populations involved in autoimmune inflammation	Effective in diseases like rheumatoid arthritis, psoriasis, and autoimmune diseases	Orencia	2005–2021	2007	Rheumatoid arthritis, juvenile idiopathic arthritis, psoriatic arthritis, graft-versus-host disease prophylaxis	Infection, nausea, nasopharyngitis, headache	[66,67,75,76]
	Alefacept—fusion protein targeting CD2-mediated T-cell activation			Amevive	2003–DISCONTINUED	-	Previously approved for psoriasis; later discontinued		

**Table 4:** Small-Molecule Inhibitors for Representative Autoimmune and Chronic Inflammatory Diseases.

Inhibitor Class	Target	Mechanism of Action	Current Status	Example Agents	References
JAK Inhibitors	JAK1, JAK2, JAK3, TYK2	Inhibition of Janus kinase-mediated intracellular inflammatory signalling	Approved and in clinical use	Tofacitinib, Baricitinib, Upadacitinib	[100,104,109]
BTK Inhibitors	Bruton’s Tyrosine Kinase (BTK)	Kinase inhibition affecting B-cell receptor signalling	In clinical trials	Ibrutinib, Acalbrutinib	[127]
SYK Inhibitors	Spleen Tyrosine Kinase (SYK)	Kinase inhibition affects immune-cell activation and inflammatory signalling	In clinical trials	Fostamatinib	[128]
S1P Receptor Modulators	Sphingosine 1-phosphate receptor (S1PR)	Selective receptor modulation affecting immune-cell trafficking	Approved and in clinical use	Fingolimod, Ozanimod	[129,130,131]
PDE4 Inhibitors	Phosphodiesterase 4 (PDE4)	Enzyme inhibition leading to reduced inflammatory signalling	Approved and in clinical use	Apremilast	[132,133]

**Table 5:** Cell-Based and Gene Therapies for Representative Autoimmune and Chronic Inflammatory Diseases.

Therapy	Mechanism of Action	Clinical Applications	Main Challenges
Mesenchymal Stem Cells (MSCs)	Immunomodulation, tissue repair and regulation of inflammatory immune-cell responses	RA, SLE, Crohn’s disease, and MS,	Limited long-term efficacy, variability in donor cells and inconsistent durability of response
CAR-T Cell Therapy	T-cell modification to target specific antigens	Selected therapy-resistant autoimmune diseases with B-cell involvement, including SLE	Risk of cytokine release syndrome, high cost, limited availability
Treg Cell Therapy	Expansion of regulatory T cells to restore immune tolerance	RA, SLE, T1D, and MS	Difficulty in expanding functional Tregs, long-term stability issues and limited clinical evidence
CRISPR Gene Editing	Precision editing of immune system genes	Potential for genetic correction of autoimmune disorders	Ethical concerns, delivery challenges, potential off-target effects and limited human clinical data

**Table 6:** Microbiome-Based Therapeutic Strategies in Representative Chronic Inflammatory and Autoimmune Diseases.

Therapy	Mechanism of Action	Impact on Inflammation	Impact on Inflammation (Cells/Cytokines)	References
Probiotics	Live microbes that promote gut and immune health	Modulate immune system response, reduce systemic inflammation	Increase Treg cell activity, decrease proinflammatory cytokines (e.g., IL-6, TNF-α), enhance Th1/Th2 balance	[180]
Faecal Microbiota Transplantation (FMT)	Transfer of faecal matter from healthy donors to patients	Rebalances gut microbiota, alleviates dysbiosis-induced inflammation	Restores balance of Th1/Th17 cells, decreases inflammatory cytokines (e.g., IL-17, IL-23), enhances Treg function, reduces IL-1β and TNF-α	[181]
Microbiome-Modulating Drugs	Drugs that modify gut microbiota composition (e.g., prebiotics, postbiotics)	Regulate gut immune responses, influence systemic inflammatory processes	Regulate macrophage polarisation, increase anti-inflammatory cytokines (e.g., IL-10), suppress inflammatory cytokines (e.g., IL-1β, TNF-α)	[182,183]

**Table 7:** Nanotechnology-Based Drug Delivery Systems for Representative Autoimmune and Chronic Inflammatory Diseases.

Nanocarrier	Mechanism of Action	Advantages in Drug Targeting for Immune Disorders	Example Drugs	Clinical Trial	Safety Profile	References
Liposomes	Lipid bilayer vesicles that encapsulate drugs	Enhanced drug stability, improved bioavailability, targeted delivery to immune cells, reduced toxicity	Doxorubicin Methotrexate	In Cancer therapies, for RA	Injection site reactions	[188]
Nanoparticles	Small particles that can be functionalised with ligands	High surface area for drug loading, ability to cross biological barriers, targeted delivery to specific tissues or cells	Hydroxychloroquine Tacrolimus	Nanoparticle delivery systems in SLE, RA	Varies by formulation, potential for immune responses	[189]
Exosomes	Biologically derived vesicles from cells	Natural biocompatibility, ability to carry proteins, RNA, and other therapeutic agents, targeted immune modulation.	Rituximab	Exosome-based therapies for IBD and RA	Require further safety data	[190]
mRNA Vaccines (LPN-based)	Lipid nanoparticles encapsulating modified mRNA antigenic proteins	Efficient in in vivo translation; induction of adaptive immunity; potential in autoimmune regulation	COVID-19 mRNA	Personalised vaccines for IBD	Long-term safety monitoring is ongoing	[191]
Dendrimers	Synthetic polymers for multivalent drug delivery	Targeted interaction with immune cells; high drug-loading capacity	Methotrexate	-	-	[192]
Micelles	Amphiphilic molecules form nano-sized spherical structures	Improve solubility of hydrophobic drugs, passive targeting via EPR effects, low immunogenicity	Cyclosporine A	-	-	[193]

**Table 8:** Ongoing Clinical Trials for Novel Therapies.

Trial Name	ClinicalTrials.gov ID	Drug Candidate	Target Disease	Phase	Primary Outcomes	Estimated Completion
BAH247	NCT06428188	BCMA/CD19 CAR-T cells	Systemic Lupus Erythematosus, Sjögren’s Syndrome	Phase 1/2	Incidence and severity of dose-limiting toxicities	December 2026
CASTLE	NCT06347718	Anti-CD19 CAR-T cell therapy	Systemic Lupus Erythematosus, Systemic Sclerosis, Dermatomyositis, Polymyositis	Phase 1/2	Safety assessment (CRS and ICANS severity)	May 2026
CRI-RA	NCT04870203	Baricitinib + Anti-TNF therapy	Rheumatoid Arthritis	Phase 3	ACR50 response rate, DAS28-CRP change	December 2026
FARGO	NCT06286709	Faecal Microbiota Transplant	Primary Sclerosing Cholangitis, Inflammatory Bowel Disease	Phase 2a	Reduction in serum ALP values	July 2026
Autologous Stem Cell Transplant for Crohn’s Disease	NCT03219359	Autologous Stem Cell Transplant + Cyclophosphamide + Vedolizumab	Crohn’s Disease	Phase 2	Proportion of patients in clinical remission (CDAI <150)	April 2026
U-EMPOWER	NCT06332534	Upadacitinib	Crohn’s Disease (Paediatric)	Phase 3	Clinical response per PCDAI at Week 12, clinical remission at Week 64, endoscopic response at Week 64	December 2034
SELECT-SLE	NCT05843643	Upadacitinib	Systemic Lupus Erythematosus	Phase 3	BICLA response rate	October 2027
RA-PRO PRAGMATIC TRIAL (RA-PROPR)	NCT04692493	Targeted synthetic DMARD, non-TNFi-biologic	Rheumatoid Arthritis	Phase 3	Functional limitation (HAQ score)	December 2028
POSTERITY	NCT05039619	Obinutuzumab, Placebo, Mycophenolate Mofetil	Lupus Nephritis (Adolescents and Paediatric	Phase 2	Percentage of Participants achieving Complete Renal Response (CRR), Percentage of Participants with Adverse Events (PP)	2027
BEAT-MS	NCT04047628	Autologous Hematopoietic Stem Cell Transplant, Best Available Therapy	Relapsing Multiple Sclerosis, Relapsing Remitting Multiple Sclerosis, Secondary Progressive Multiple Sclerosis	Phase 3	MS relapse-free survival (time from randomisation until MS relapse or death from any cause)	October 2029
TARA	NCT06020144	TLL-018, Tofacitinib	Rheumatoid Arthritis	Phase 3	ACR50 response rate (≥50% improvement in joint counts and at least 3 of 5 remaining ACR core measures)	September 2025
INSPIRE 1	NCT04314544	Tildrakizumab, Placebo	Active Psoriatic Arthritis	Phase 3	Proportion of subjects achieving ACR20 (≥20% reduction from Baseline in response criteria)	February 2026

## Data Availability

No new data were created or analysed in this study.
